# Developing and validating a genre awareness questionnaire for writing scientific reports

**DOI:** 10.3389/fpsyg.2023.1210240

**Published:** 2023-06-29

**Authors:** Jack Pun, Kason Ka Ching Cheung

**Affiliations:** ^1^Department of English, City University of Hong Kong, Hong Kong, Hong Kong SAR, China; ^2^Department of Education, University of Oxford, Oxford, United Kingdom

**Keywords:** genre awareness, L2 writing, scientific reports, IMRD genres, English medium instruction (EMI)

## Abstract

Current scholarship on language awareness focuses on learners' noticing of forms and functions of language. In writing scientific reports, learners need to be aware of the Introduction, Method, Results, and Discussion (IMRD) genres. While scholarship explores students' language awareness in writing genres for scientific reports, there is a limited quantitative instrument for researchers to measure students' language awareness in these four genres of writing scientific articles. This study investigates the structure of factors of Language Awareness of Genres in a Scientific Writing Questionnaire that measures students' awareness of IMRD genres for L2 bilingual secondary school students in Hong Kong (*N* = 234). Content validity and construct validity were used. The results show that this instrument is reliable for researchers and teachers to understand the effectiveness of specific genre-based interventions on genre awareness.

## Introduction

Promoting language skills and expression of language is an apparent objective in all subjects in K-12 education, including science. This presents particular challenges for English-as-a-second-language (ESL) learners, as they need to bridge meanings across their first language (L1) and second language (L2) (Pun et al., 2023). Current scholarship focuses on micro-analysis of what difficulties students face when they write technical terms [e.g., Ha and Hyland (2017)], non-technical terms (e.g., Coxhead, 2000), or use logical connectives (Quílez, 2021). What is more important is that learners need to recognize writing scientific texts as a staged and goal-oriented process, becoming aware of the structures and purpose of the activity (Tang, 2022). Scientific genres are more than single lexicons or grammars, as they comprise the structure and organization of texts (Tang, 2022). While there is scholarship of examining the types of genres in school science (Tang et al., 2022), there are limited quantitative research studies on how we can measure K-12 students' awareness of these scientific genres.

To measure the success of a genre-based intervention in developing ESL learners' awareness of genres of writing scientific reports, there is a need for a validated instrument to measure such effectiveness. By understanding ESL learners' language awareness in writing scientific reports, the link between their epistemological motivation and their ability to write different scientific report genres could be thoroughly investigated by educators and researchers (Kress et al., 1998). ESL learners' language awareness in scientific writing varies according to the Introduction-Method-Results-Discussion (IMRD) genres (Hyland, 1996). There are customary purposes and functions for each IMRD genre which students need to be aware of. As initial steps, the purpose of the current study was to design and validate an instrument that measures ESL learners' awareness of IMRD genres in writing scientific reports.

## Literature review

### Language awareness in scientific writing

Language awareness is defined as “explicit knowledge about language, and conscious perception and sensitivity in language learning, language teaching, and language use” (Svalberg, 2012: 376). It can be constructed through students' and teachers' joint engagement with language (Svalberg, 2007). According to Eckerth (2008), language awareness is a kind of explicit knowledge that affects how knowledge about writing is processed and turned into output writing. In a review study by Svalberg (2016), language awareness places exclusive focus on language structure such as grammar and lexis. However, Leonard (2013, p. 168) argues that language outcomes are not measured by “proficiency […] as a level of acquisition but as a stage in attunement” which involves “language negotiation and play with specific audiences in certain stations.” In other words, learners should recognize the purposes and functions of language in a specific context.

Despite the central role of language awareness in writing, studies either investigated classroom episodes of how teachers develop students' language awareness in writing scientifically (Seah and Yore, 2017; Seah and Silver, 2020) or the effectiveness of a genre-based approach in improving students' writing (Ellis et al., 1998). As secondary students are novices to scientific report writing, students' writing competence in scientific reports might not be the only indicator of the learning outcome of genre-based intervention because the focus on their acquisition of genres only teaches them to reproduce texts from a template. Instead of genre acquisition, a focus on students' awareness of scientific report genres helps students to develop “the rhetorical flexibility necessary for adapting their sociocognitive genre knowledge to ever-evolving contexts”. Genre-based intervention should not merely focus on students' acquisition of genres in scientific reports but it should also emphasize their genre awareness in writing scientific reports (Kelly-Laubscher et al., 2017). Therefore, with a focus on both genre writing and genre awareness in teaching scientific writing, students would not replicate the lexicogrammar structures from templates and master the skills of crafting written language according to the purposes and functions of the dynamic scientific contexts.

### Instruments for measuring genre awareness in scientific writing

In response to the call of promoting students' language awareness of using genre in scientific writing, there is a need for an instrument available to measure the effectiveness of genre-based intervention. Genre awareness, which is a subset of language awareness, refers to students' development of rhetorical flexibility to adapt their knowledge of socio-cognitive genres in changing contexts (Freedman, 1993). In completing a scientific writing task, learners need to be aware of the purpose, context, writer's role, audience, content, and sources of the task.

To investigate students' awareness of their rhetorical flexibility, instruments such as self-reported agreement scales (e.g., Hedgcock and Lee, 2017; Crosthwaite et al., 2021) and test performance (e.g., Zare and Keivanloo-Shahrestanaki, 2017) were developed. However, genre awareness is disciplinary-specific. For example, in the discipline of life science, the genre concerns more with explanation and methodological account; while in the discipline of social science, the genre concerns more with case studies and problem questions (Crosthwaite et al., 2021). As a result, previous instruments cannot be applied to measure students' genre awareness in scientific writing, as these instruments were devised from a different discipline of study.

### “IMRD” genre awareness in scientific writing

Conscious writing facilitates learners to acquire explicit knowledge of the target language and linguistic features (Takimoto, 2006; Eckerth, 2008). As argued in previous sections, intervention should aim at improving ESLs' awareness of how linguistic features are structured according to the purpose of an activity (Martin and Rose, 2008).

The genre-based approach helps students develop their awareness of the macrostructures of writing scientific reports. A genre-based approach improves students' use of conjunctions and contains obligatory moves (Ellis et al., 1998). In school science, scientific genres were defined as comprising experimental accounts, information reports, explanations, and arguments (Tang, 2022; Tang et al., 2022). However, in writing authentic scientific reports, secondary school students need to be aware of semantic variations in Introduction, Method, Results, and Discussion (IMRD) prototypical genres (Swales, 1990). O'Neill argued that there are customary argument functions for each IMRD genre which we argue that students need to be aware of:

- Genre Awareness for writing *Introduction*: recognizes the importance of outlining the purpose, question, and the issue of a problem, explaining the importance of the purpose, summarizing methods and results, providing background research, and summarizing key findings;
- Genre Awareness for writing *Method*: recognizes the importance of describing what is done by researchers and explaining how the methods can answer the research question;
- Genre Awareness for writing *Results*: recognizes the importance of foreshadowing results, presenting brief data, interpreting data in relation to the problem, and communicating data to non-specialists;
- Genre Awareness for writing *Conclusion*: recognizes the importance of stating the conclusion relevant to the original question, describing whether the data accept or refute the hypothesis, discussing the significance of the question, and making the suggestion for further study.

Compared to the approach by Tang et al. (2022), using the IMRD approach is a shared practice in science classrooms and authentic scientific research (Li and Flowerdew, 2020). Authentic scientific research articles, which adopt the IMRD prototype, share similarities with the structures of scientific reports in school science (Jackson et al., 2006). Roo and Ardasheva also argued that an IMRD approach aligns with the process of scientific research in school science. However, students do not necessarily develop an awareness of each genre of IMRD to the same extent. For example, pre-adolescents struggle to develop their awareness in Discussion because they are not often exposed to expository texts (Fang, 2005). Factors such as variation between different grades of students in gaining awareness in writing a specific genre were observed in Fang study.

## Method

### Developing the items of the IMRD genre awareness instrument

To develop the instrument of learners' awareness of IMRD genres, we searched the literature for conceptual frameworks that can address the features of IMRD genres in university and K-12 science writing. An instrument was developed based on the IMRD features by Kelly-Laubscher et al. (2017). Two academics who research second language writing and another academic whose expertise is in secondary science education reviewed the instrument, ensuring that the theoretical content of the items aligns with the framework of Kelly-Laubscher et al. (2017). Together with the three academics, three research assistants who have teaching and research experience in EMI science classrooms from the expert pool rated whether each item has measured the targeted construct and can be understood by secondary school students. In this process, the research team reviewed the items according to some curriculum standards from EMI science classrooms. The reviewers removed items that were rated as low face validity.

The version of the developed instrument, reported in this study, resulted in a 30-item questionnaire, learner-focused and criterion-referenced, which addressed the awareness of special purposes and functions of each IMRD genre in writing scientific reports (Appendix). The original instrument comprised four components that dealt with the purposes and functions of Introduction (3 items), Method (7 items), Results (11 items), and Discussion (9 items). The scale ranged from 1, corresponding to “strong disagree,” to 5, corresponding to “strongly agree.” After confirming the theoretical justification of the items in the questionnaire, it was then administered to secondary school students.

### Participants

The first author sent an invitation to schools in Hong Kong. Eight schools volunteered to participate in this teaching project. A total of 234 Hong Kong secondary school students (male: 112; female: 122) participated in a project of “Genre-based Approach in Improving Scientific Writing.” Data were collected in a project that uses a genre-based approach to improve genre awareness and competence in scientific writing. Our sample consists of junior form (Grade 7 to 9) students (*n* = 170, 72.6%) and senior form (Grade 10–11) students (*n* = 61, 26.1%). Three students did not answer the question regarding grade levels; 217 (92.7%) of the participants reported that their first language is Cantonese. Our sample also consisted of participants with a first language other than Cantonese, for example, English (*n* = 7, 3.00%), Dutch (*n* = 1, 0.04%), Nepali (*n* = 1, 0.04%), and Mandarin (*n* = 8, 3.42%). In Hong Kong, most students are multilingual as they must learn Cantonese, Mandarin, and English in primary and secondary school curricula.

### Establishing construct validity using confirmatory factor analysis

Construct validity of the IMRD genre awareness instrument was established by confirmatory factor analysis, using the software of Mplus 8.3. According to Knapp and Mueller, construct validity can demonstrate the extent of agreement between the questionnaire items and theoretical foundations. Confirmatory factor analysis (CFA) shows the extent of agreement of the questionnaire items with the IMRD genre awareness (Harrington, 2009), based on the conceptual framework by Kelly-Laubscher et al. (2017). In the original version of the instrument, one author from an applied linguistics background and another author from a science education background collaborated in drafting the factorial constructs (Genre awareness of Introduction, Method, Results, and Discussion) based on reviewing prior literature (Kelly-Laubscher et al., 2017). Prior literature focuses on conceptualizing the application of IMRD genres in undergraduate education (Storey, 2013). There is a limited number of studies that delineates the frameworks of IMRD genres in school science report writing. Therefore, we adopted these definitions and drafted the items such that they are applicable to school science level. The instrument was then reviewed by three science teachers. However, to evaluate the psychometric properties of these constructs of genre awareness in writing scientific reports, CFA can provide an evaluation of the instrument's construct validity and help authors decide on items that have low factor loadings (Brown, 2015).

The univariate and multivariate normality of each item was checked by inspecting the skewness and kurtosis of each item. Maximum likelihood (ML) was used to provide parameter estimates and their standard errors (Yuan and Bentler, 2000). Data-model-fit indices were used to inspect the construct validity of each parameter (i.e., awareness of IMRD genre). Four indices were used to examine the construct validity: (1) Comparative Fit Index (CFI), (2) Tucker–Lewis Index (TLI), (3) Standardized Root Mean Square Residual (SRMR), and (4) Root Mean Square Error of Approximation (RMSEA) (West et al., 2012). As recommended by Brown and Cudeck (1992), Hu and Bentler (1999), and Marsh et al. (2004), the cutoff criteria of model fit indices are CFI ≥ 0.95, TFI ≥ 0.95, SRMR ≤ 0.08, and RMSEA ≤ 0.06. Convergent validity of each construct was investigated by computing Composite Reliability (CR) and Average Variance Extract (AVE). Compared to Cronbach's Alpha, CR is a less biased indicator of the shared variance between latent variables of the model, while AVE captures the level of variance of a construct compared to the level owing to measurement error (Alarcón et al., 2015). An acceptable convergent validity of each construct can be evidenced by CR ≥ 0.7 and AVE ≥ 0.5.

### Testing in a pilot teaching intervention

We also hypothesize that this instrument can measure the change in outcomes of teaching intervention. The instrument was also administered before and after a pilot genre-based teaching intervention. In the genre-based teaching intervention, the teachers worked with their students through a sequence of five steps in the teaching-and-learning cycle (Rose and Martin, 2012). These steps were (1) developing the students' basic understanding of their subject and of the social context in which the genre operates; (2) carefully analyzing sample texts as a way of developing the student's comprehension of the communicative purposes that the texts serve, and understanding the language choices used to serve those purposes; (3) constructing texts jointly, with the teachers acting as disciplined language guides; (4) constructing texts individually, with each student demonstrating his or her capability; and (5) reviewing the students' texts to provide input for subsequent teaching and learning cycles. Week 1 of the 12-week intervention gave an overview of scientific research. Weeks 2 and 3 introduced the assigned topics. Weeks 4–9 explored the organization of scientific reports, which included the purposes of each section of an IMRD paper and how to integrate research findings into a discussion. In weeks 10–12, students drafted their reports. The teachers received the same set of teaching materials. One of the teachers was asked to use more interactive activities (a dialogic approach) and to present the materials in such a way that the genre features of the scientific texts were made explicit to students. The other teacher followed the same procedures but did not implement interactive activities. However, owing to the COVID-19 pandemic, only 92 students completed both instruments before and after the intervention.

## Results

### Item summary statistics

The mean, standard deviation, and skewness of kurtosis for all items in the instrument were inspected to ensure univariate normality. Univariate normality should be assessed before running CFA (Bandalos and Finney, 2010). Items with acceptable normality have a skew and kurtosis in the range between −2 and +2. According to Table 1, all items have skewness and kurtosis falling into the range, meaning that all items follow univariate normality.

**Table 1:** Item summary statistics.

**Factor**	**Items**	**Mean**	**Standard deviation**	**Skewness**	**Kurtosis**
Genre awareness for Introduction (GAI)	1. I know the three purposes of an introduction (writers identify the topic, explain why it's important, state the purpose of the study).	3.590	0.7601	−0.159	0.034
	2. I know that the end of introduction describes the specific aims of the study.	3.545	0.7549	−0.252	0.045
	3. I know the basic functions of an introduction (a. identify a worthwhile research area; b, identify a topic within that area which needs more research; c. tell your reader what you're going to do about it).	3.594	0.7652	−0.089	−0.023
Genre awareness for Method (GAI)	4. I know the purpose of method section is to tell what the researcher did.	3.615	0.7845	−0.280	0.002
	5. I know how to write a good method section.	3.269	0.7585	−0.197	−0.100
	6. I know that the purpose of a method section is to describe how the research is carried out.	3.560	0.7687	−0.260	0.016
	7. I know a method section although the content is varied according to the topic, it asks 1) what kind of data were used; 2) how were they collected; 3) how were they analyzed.	3.509	0.7709	−0.199	0.243
	8. I know that a good method section can help readers judge how convincing they find the results; and help researchers who want to reproduce or adapt the study.	3.536	0.7995	−0.355	0.382
	9. I know that a method section should explain why the chosen procedures were used are appropriated.	3.464	0.7722	−0.167	0.180
	10. I know that method section tells a story about research that you carried out in the past, with emphasis on the procedures rather the people who are doing them.	3.491	0.7806	−0.012	−0.129
Genre awareness for Results (GAR)	11. I know that the result section tells what the researchers learned.	3.737	0.7698	−0.211	0.313
	12. I know how to write a good result section.	3.280	0.7638	−0.052	0.362
	13. I know that the section heading of result section may say something other than result such as findings. Sometimes result and discussion sections are combined.	3.438	0.7816	−0.126	−0.174
	14. I know that result section presents the findings verbally and using range of tables, figures, illustrations or textual examples.	3.528	0.7836	−0.099	0.163
	15. I know that results are typically expressed in the past tense.	3.667	0.8274	−0.211	−0.021
	16. I know I need to use present tense when findings are related to things continue to be true.	3.752	0.7946	−0.283	0.225
	17. I know that result section addresses the research hypothesis or the expectations about what the results could be.	3.558	0.7737	−0.370	0.558
	18. I know that words or phrases related to discovery, learning or understanding are often used in result section.	3.605	0.7286	−0.277	0.173
	19. I know that hedging (i.e. limiting how strongly or confidently express yourself) is often used in result section.	3.378	0.8203	−0.033	0.076
	20. I know that an efficient way of presenting data is figure, table and illustration.	3.673	0.7841	−0.244	0.226
	21. I know that result section describes the data that study has produced, words and graphics and does not go into details of the findings.	3.378	0.7492	−0.048	−0.113
Genre awareness for Discussion (GAD)	22. I know that the discussion section puts things in context and tells what the results mean.	3.489	0.8292	−0.199	−0.142
	23. I know how to write a good discussion section.	3.259	0.8093	0.000	0.257
	24. I know that the main purpose of a discussion section is to explain significant results.	3.579	0.7606	−0.278	0.332
	25. I know that one approach to writing a discussion section is to refer to research questions, hypothesis or objectives which was initially presented in the introduction section.	3.404	0.7754	−0.156	0.044
	26. I know that the second approach to writing a discussion section is to highlight the principal findings.	3.412	0.7819	−0.039	0.074
	27. I know that I need to be selective when writing up findings in discussion and do not repeat all what you have mentioned.	3.524	0.7919	−0.006	0.061
	28. I know that the third approach to writing a discussion section is to explain how your findings are related to early research.	3.451	0.8015	−0.173	0.217
	29. I know a range of strategies in writing discussion such as: confirming existing knowledge of the topic, or challenging early research or drawing attention to the results that are new or suggesting an explanation to explain a finding; identifying quest.	3.335	0.7581	−0.151	0.672
	30. I know that discussion highlights the significance of the findings.	3.613	0.7841	−0.414	0.586

### Construct validity

The final four-factor model for measuring genre awareness in scientific report writing reached the cutoff criteria of construct validity and convergent validity. As argued, each type of genre has its own purpose and function and is characterized by its lexicogrammar in writing scientific reports. These items comprise statements related to the purposes, functions, and choices of lexicons of each IMRD genre (Kelly-Laubscher et al., 2017). For each construct, we examined indices for convergent validity (CR and AVE) and construct validity (CFI, TFI, RMSEA, and SRMR). In case of unsatisfactory model-fit indices, we removed items that have the lowest factor loadings. Table 2 indicates the model-fit indices as well as the factor loadings of each construct.

**Table 2:** Instrument item and factor loading.

**Item**	**GAI**	**GAM**	**GAR**	**GAD**	**Composite Reliability (*CR*)**	**Average Variance Extracted (*AVE*)**
**Genre Awareness of Introduction (GAI)**					
1. I know the three purposes of an introduction (writers identify the topic, explain why it's important, state the purpose of the study).	0.695				0.821	0.606
2. I know that the end of introduction describes the specific aims of the study.	0.826					
3. I know the basic functions of an introduction (a. identify a worthwhile research area; b, identify a topic within that area which needs more research; c. tell your reader what you're going to do about it).	0.807					
**Genre Awareness of Method (GAM)**					
7. I know a method section although the content is varied according to the topic, it asks 1) what kind of data were used; 2) how were they collected; 3) how were they analyzed.		0.726			0.881	0.651
8. I know that a good method section can help readers judge how convincing they find the results; and help researchers who want to reproduce or adapt the study.		0.825				
9. I know that a method section should explain why the chosen procedures were used are appropriated.		0.857				
10. I know that method section tells a story about research that you carried out in the past, with emphasis on the procedures rather the people who are doing them.		0.813				
**Genre Awareness of Result (GAR)**					
11. I know the result section tells what the researchers learned.			0.787		0.924	0.604
13. I know that the section heading of result section may say something other than result such as findings. Sometimes result and discussion sections are combined.			0.720			
14. I know that result section presents the findings verbally and using range of tables, figures, illustrations or textual examples.			0.765			
15. I know the results are typically expressed in past tense.			0.732			
16. I know I need to use present tense when findings are related to things continue to be true.			0.772			
17. I know that result section addresses the research hypothesis or the expectations about what the results could be.			0.838			
19. I know that hedging (i.e. limiting how strongly or confidently express yourself) is often used in result section.			0.793			
20. I know an efficient way of presenting data is figure, table and illustration.			0.821			
**Genre Awareness of Discussion (GAD)**					
22. I know that the discussion section puts things in context and tells what the results mean.				0.812	0.944	0.727
24. I know that the main purpose of a discussion section is to explain significant results.				0.824		
25. I know that one approach to writing a discussion section is to refer to research questions, hypothesis or objectives which was initially presented in the introduction section.				0.861		
26. I know the second approach to writing a discussion section is to highlight the principal findings.				0.828		
27. I know that I need to be selective when writing up findings in discussion and do not repeat all what you have mentioned.				0.778		
28. I know that the third approach to writing a discussion section is to explain how your findings are related to early research.				0.864		
29. I know a range of strategies in writing discussion such as: confirming existing knowledge of the topic, or challenging early research or drawing attention to the results that are new or suggesting an explanation to explain a finding; identifying quest.				0.788		
30. I know that discussion highlights the significance of the findings.				0.832		
**Fit Indices**					
CFI	1.000	1.000	0.982	0.983		
TFI	1.000	1.000	0.975	0.976		
RMSEA	0.000	0.008	0.066	0.074		
SRMR	0.000	0.000	0.024	0.023		

To test the strength of partial correlation, a Kaiser–Meyer–Olkin (KMO) Measure of Sampling Adequacy of 0.963 was obtained. Bartlett's Test of Sphericity (χ^2^ = 6109.458, df = 435, *p* < 0.01) justifies that variables are related and ideal for factor analysis.

Genre awareness of introduction (GAI) in writing scientific reports concerns the purpose and functions of writing a scientific introduction, for example, item 2 “I know that the end of introduction describes the specific aims of the study” and item 3 “I know the basic functions of an introduction (a. identify a worthwhile research area; b, identify a topic within that area which needs more research; c. tell your reader what you're going to do about it).” The factor loadings of the items range from 0.695 to 0.826. The item “I know the three purposes of an introduction (writers identify the topic, explain why it's important, state the purpose of the study)” has a factor loading slightly lower than 0.7. However, this item describes the structure of the genre of introduction; therefore, this item was kept in our version of the instrument. The construct capturing GAI yielded CR of 0.821 and AVE of 0.606, while CFI and TFI of 1.000 and RMSEA and SRMR of 0.00. These indices indicate that the items measuring GAI have an acceptable model fit.

Meanwhile, for genre awareness of writing scientific method (GAM), we removed item 4, item 5, and item 6 as these items have a factor loading way below 0.7. A factor loading of 0.7 indicates a higher factor loading (Brown, 2015). The remaining items, items 7 to 10, capture students' awareness of the function of the scientific method genre. The factor loadings of these four remaining items range from 0.726 to 0.857. Construct validity and convergent validity of this construct of genre awareness were also examined. Indices for convergent validity (CR = 0.881, AVE = 0.651) and construct validity (CFI = 1.000, TFI = 1.000, RMSEA = 0.008, SRMR = 0.000) indicate that the four items are a perfect fit for the construct of GAM.

For genre awareness of reporting scientific results (GAR), we removed item 12 as it has a factor loading below 0.7. After the removal of item 12, the model fit indices still did not attain an acceptable fit according to the cutoff criteria of Hu and Bentler (1999). Until the removal of two items with comparatively lower factor loadings (items 18 and 21), the construct has a satisfactory convergent validity (CR = 0.924, AVE = 0.604) and construct validity (CFI = 0.982, TFI = 0.975, RMSEA = 0.066, and SRMR = 0.024). These construct items comprise an examination of students' awareness of using correct tenses, hedging devices, purposes, and functions in the results genre. A sample item, item 19 “I know that hedging (e.g., limiting how strongly or confidently express yourself) is often used in result section” concerns the use of hedging devices in expressing the validity of the results of scientific investigation.

**Figure 1:**
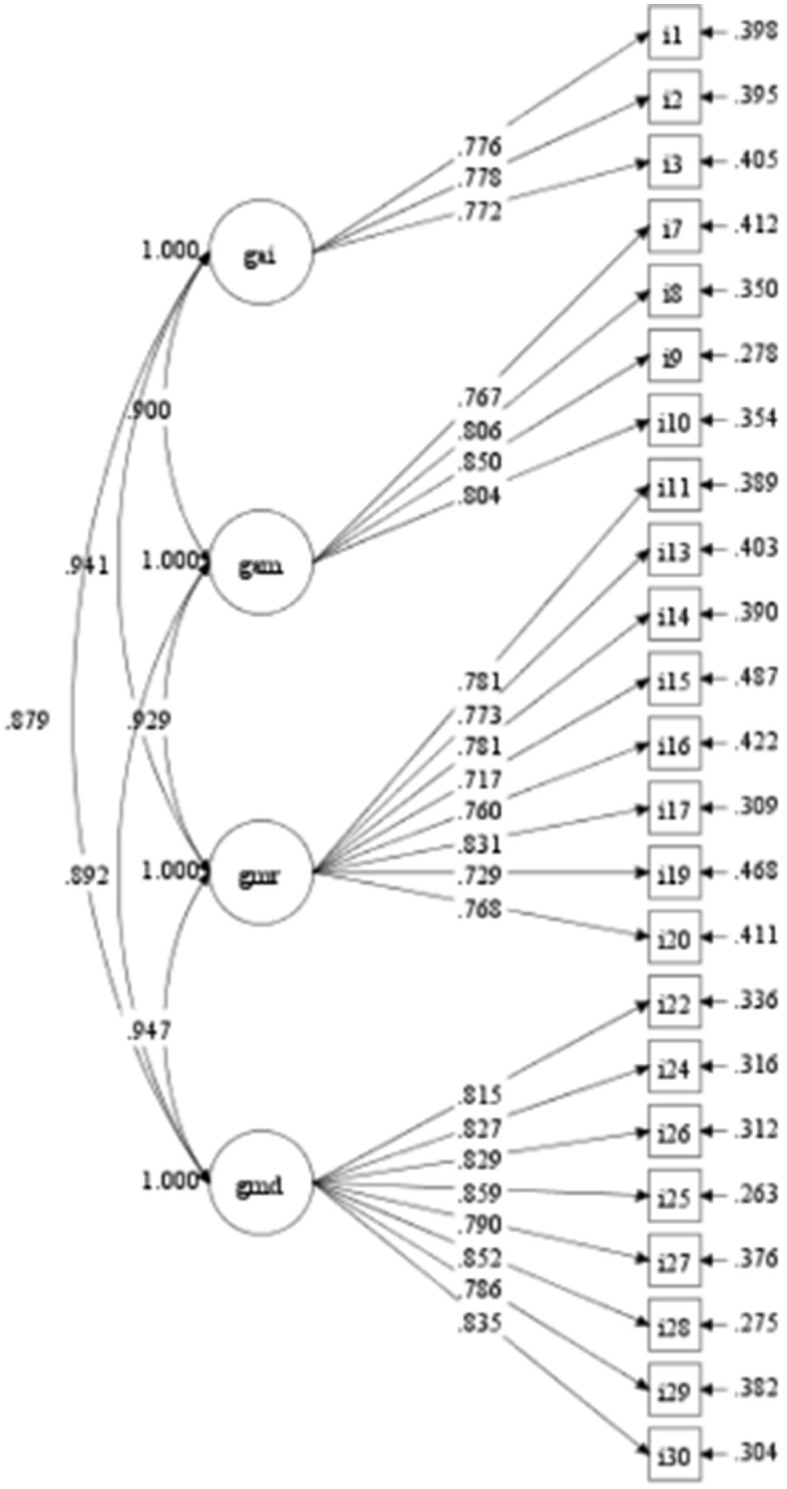
Finalized model and parameter estimates from CFA validation data. Item number corresponds those listed in Appendix.

For genre awareness of writing discussion in a scientific report (GAD), we removed item 23 in order to attain satisfactory model fit indices. The remaining seven items (items 22, 24–30) have factor loadings above 0.7 which range from 0.778 to 0.861. Construct validity and convergent validity of this construct of genre awareness were also examined. Acceptable indices for convergent validity (CR = 0.944 and AVE = 0.727) and construct validity (CFI = 0.983, TFI = 0.976, RMSEA = 0.074, and SRMR = 0.023). A finalized model was created (Figure 1), and the correlations between four IMRD constructs were investigated. All items yielded a factor loading above 0.7, indicating the items are loaded into the construct very well. The final version of the 23-item instrument consists of 3 items for GAI, 4 items for GAM, 8 items for GAR, and 8 items for GAD.

### Piloting in a teaching intervention

Paired samples *t*-tests were performed on students' GAI, GAM, GAR, and GAD. According to Table 3, student self-efficacy on all constructs of genre awareness is significantly higher in the post-test compared to pre-test under a pilot genre-based intervention: GAI increases significantly by 0.82 [*t*_(91)_ = −2.798, *p* = 0.006]; GAM increases significantly by 0.35 [*t*_(91)_ = 4.328, *p* < 0.001]; GAR increase significantly by 0.25 [*t*_(91)_ = 3.690, *p* < 0.001]; GAD increase significantly by 0.31 [*t*_(91)_ = 3.934, *p* < 0.001]. This shows that the instrument is effective in measuring the outcomes of genre-based intervention.

**Table 3:** Results of *t*-tests on all students' GAI, GAM, GAR, and GAD.

**Components of scientific writing**	**Pretest**		**Posttest**		***t*** **-test**	
	***M***	***SD***	***M***	***SD***	***p***	***Cohen's d***
GAI	3.59	0.58	3.77	0.62	0.006^**^	0.292
GAM	3.53	0.67	3.88	0.65	< 0.001^***^	0.451
GAR	3.67	0.62	3.92	0.64	< 0.001^***^	0.385
GAD	3.53	0.70	3.84	0.63	< 0.001^***^	0.410

^**^p ≤ 0.01, ^***^p ≤ 0.001.

## Discussion and conclusion

The four-factor model for the IMRD genre awareness instrument showed very good model fit and sub-scale reliabilities. The model itself is also aligned with the theoretical framework from Kelly-Laubscher et al. (2017). In other words, this instrument can be administered as a large-scale survey to measure the effectiveness of genre-based intervention on secondary school students' genre awareness in writing scientific reports.

First, content validity was ensured by writing the items on a strong theoretical basis of IMRD genre literature. A thorough review of the literature (Kelly-Laubscher et al., 2017) also justified the theoretical constructs captured by the items in the IMRD genre awareness instrument. This instrument has been reviewed by researchers from various disciplines such as applied linguistics and science education. It has also been authenticated by members of the research team who have substantial teaching and college experiences. To ensure that this instrument can be used to measure the effectiveness of a pilot genre-based teaching intervention, we applied this instrument in a pilot teaching intervention and compared the results of students with different grade levels (e.g., junior form students and secondary form students).

Genre awareness is a critical issue that helps students develop their flexibility in applying different features of purposes, functions, and lexicons in ever-evolving contexts. The focus of this research was to develop and provide an available instrument for researchers to measure the awareness of IMRD genres. The validity of this self-report questionnaire survey was supported, and we used different model fit indices to establish the creditability of this questionnaire. It provides teachers to gather information on which constructs of genre awareness have been improved under an explicit focus on the genre in scientific writing. More importantly, researchers can use this instrument to examine the relationships between different constructs of genre awareness and their writing scores, as well as evaluate teaching intervention programs. As demonstrated in the results of our pilot intervention, this instrument can measure the change in different constructs of genre awareness in writing scientific reports. This shows that students' awareness of different genres is differentially improved under a particular teaching intervention programme.

The limitation of this instrument is that it is only based on IMRD genres. IMRD genre was chosen because it reflected the authentic genres of practicing scientists (Swales, 1990). We also noted that there are other classifications of genre in school science such as experimental account, information report, explanation, and argument (Tang, 2022; Tang et al., 2022). There could be future research studies that develop instruments specific to these genres. The limitation of this instrument is that it has not been enriched by a qualitative study, which warrants further investigation in future studies.

## Data availability statement

The original contributions presented in the study are included in the article/supplementary material, further inquiries can be directed to the corresponding author.

## Ethics statement

The studies involving human participants were reviewed and approved by City University of Hong Kong. Written informed consent to participate in this study was provided by the participants' legal guardian/next of kin.

## Author contributions

JP contributed to the conception, design, and data collection. KC contributed to the analysis and interpretation of data. Both authors contributed to writing and revising the manuscript.

## Appendix. Original instrument measuring students' IMRD genre awareness

1. I know the three purposes of an introduction (writers identify the topic, explain why it's important, state the purpose of the study)
2. I know that the end of introduction describes the specific aims of the study.
3. I know the basic functions of an introduction (a. identify a worthwhile research area; b, identify a topic within that area which needs more research; c. tell your reader what you're going to do about it).
4. I know the purpose of method section is to tell what the researcher did.
5. I know how to write a good method section.
6. I know that the purpose of a method section is to describe how the research is carried out.
7. I know a method section although the content is varied according to the topic, it asks 1) what kind of data were used; 2) how were they collected; 3) how were they analyzed.
8. know that a good method section can help readers judge how convincing they find the results; and help researchers who want to reproduce or adapt the study.
9. I know that a method section should explain why the chosen procedures were used are appropriated.
10. I know that method section tells a story about research that you carried out in the past, with emphasis on the procedures rather the people who are doing them.
11. I know that the result section tells what the researchers learned.
12. I know how to write a good result section.
13. I know that the section heading of result section may say something other than result such as findings. Sometimes result and discussion sections are combined.
14. I know that result section presents the findings verbally and using range of tables, figures, illustrations or textual examples.
15. I know that results are typically expressed in the past tense.
16. I know I need to use present tense when findings are related to things continue to be true.
17. I know that result section addresses the research hypothesis or the expectations about what the results could be.
18. I know that words or phrases related to discovery, learning or understanding are often used in result section.
19. I know that hedging (i.e. limiting how strongly or confidently express yourself) is often used in result section.
20. I know that an efficient way of presenting data is figure, table and illustration.
21. I know that result section describes the data that study has produced, words and graphics and does not go into details of the findings.
22. I know that the discussion section puts things in context and tells what the results mean.
23. I know how to write a good discussion section.
24. I know that the main purpose of a discussion section is to explain significant results.
25. I know that one approach to writing a discussion section is to refer to research questions, hypothesis or objectives which was initially presented in the introduction section.
26. I know that the second approach to writing a discussion section is to highlight the principal findings.
27. I know that I need to be selective when writing up findings in discussion and do not repeat all what you have mentioned.
28. I know that the third approach to writing a discussion section is to explain how your findings are related to early research.
29. I know a range of strategies in writing discussion such as: confirming existing knowledge of the topic, or challenging early research or drawing attention to the results that are new or suggesting an explanation to explain a finding; identifying questions.
30. I know that discussion highlights the significance of the findings.
